# Efficacy of combination therapy with ezetimibe and statins versus a double dose of statin monotherapy in participants with hypercholesterolemia: a meta-analysis of literature

**DOI:** 10.1186/s12944-019-1182-5

**Published:** 2020-01-04

**Authors:** Min Yu, Chunshui Liang, Qianran Kong, Yihan Wang, Minmin Li

**Affiliations:** 1 Department of Oncology, The First Affiliated Hospital of Chongqing Medical University, No.1 Friendship Road, Yuanjiagang, Yuzhong District, Chongqing, 400016 China; 2 Department of Cardiovascular Surgery, Xinqiao Hospital of Army Medical University, Chongqing, 400000 China

**Keywords:** Ezetimibe, Statins, Double dose, LDL-C, Meta-analysis

## Abstract

**Background:**

The aim of this study was to compare and summarize the lipid-altering effects of combination therapy with ezetimibe and statins (E/S) and a double dose of statin (D/S) monotherapy on patients with hypercholesterolemia.

**Methods:**

We conducted search on 2 medical databases, PubMed and EMBASE to identify all relevant studies. A meta-analysis was performed to clarify the efficacy in the two groups. Only double-blind Randomized controlled study (RCTs) of efficacy evaluation in the two groups with ezetimibe and statins and a double dose of statin in participants with hypercholesterolemia that examined low-density lipoprotein cholesterol (LDL-C), total cholesterol (TC) and high-density lipoprotein (HDL) were included. Two reviewers extracted data from all primary studies independently. The primary data were the level of LDL-C, TC and HDL-C concentrations at the end point and are expressed as mean and standard deviation (SD).

**Results:**

A total of 11 double-blind, active or placebo-controlled studies with 1926 hypercholesterolemia adults randomized to ezetimibe 10 mg added to ongoing statins (*N* = 994) or statin titration (doubling) (*N* = 932) were pooled for the global meta-analysis. The effect size between treatment groups within individual studies was assessed by weighted mean difference (MD) using a random- or fixed-effect model. The result showed that the participants in E/S group get obvious lower LDL-C [MD = -13.14 mg/dL, 95%CI (−16.83, -9.44), *p* = 0.00001] and TC concentration [MD = -23.79 mg/dL, 95%CI (−38.65, -8.93), *p* = 0.002] from baseline to follow-up, comparing to the D/S group. Besides, no significant between-group differences were observed for concentrations of HDL-C [MD = 0.46 mg/dL, 95%CI (− 1.14, 2.06), *p* = 0.57]. According to subgroup analysis, the combination of ezetimibe and atorvastatin (10 mg) [MD = -16.98 mg/dL, *p* < 0 .0001] or simvastatin (20 mg) [MD = -17.35 mg/dL, *p* < 0 .0001] showed stronger ability of reducing LDL-C than combination of ezetimibe and rosuvastatin (10 mg) [MD = -9.29 mg/dL, *p* = 0.05]. The efficacy of short-term (endpoint time between 6 to 16 week) and long-term (52 week) treatment in the LDL-C between two groups did not show significant differences. Besides, only participants from Asia treated with combination therapy were associated with a significant lower LDL-C concentration [MD = -14.7 mg/dL, *p* < 0 .0001].

**Conclusions:**

The addition of ezetimibe to statin appears to be more effective on reducing LDL-C and TC concentrations than doubling the statin dose. Moreover, the ability to reduce cholesterol levels of combinations therapy with ezetimibe and different statins or to participants from different geographic location may vary, based on this meta-analysis, while more samples are needed to verify.

## Introduction

Cholesterol regulation has always been the focus of cardiovascular disease reduction in patients with coronary heart disease (CHD). Statins are the first line therapeutic approach for cardiovascular disease in patients with increased cholesterol levels or a generally increased risk of coronary heart disease [1]. Their ability to lower cholesterol and protect against CHD have been demonstrated previously [2–4]. However, some patients cannot attain LDL-C goals, even with high intensity statins [5], and those patients may experience more side effects in the liver and muscle from drug metabolism and clearance [6, 7]. High intensity statins were defined as atorvastatin 40–80 mg/d or rosuvastatin 20–40 mg/d according to the 2013 American Cardiology College/American Heart Association (ACC/AHA) guideline of cholesterol management. 2016 European Society of Cardiology/European Atherosclerotic Society (ESC/EAS) guideline recommended combination therapy with statins and other lipid-lowering drugs in cases of statin intolerance or insufficiency.

Ezetimibe has been available to low cholesterol levels as a selective cholesterol absorption inhibitor, exerting its effect through interaction with the Niemann-Pick C1- like protein 1 (NPC1L1) located in intestine. To date, numerous studies demonstrated the significant low-density lipoprotein cholesterol (LDL-C)-lowering ability and cardiovascular events prevention effect of ezetimibe-statin combination therapy [8–10]. The object of the present study was to compare the efficacy of combination therapy with ezetimibe and statin versus double-dose statin monotherapy in participants with hypercholesterolemia, reflecting the PICOS (participants, interventions, comparators, outcomes, and study design) approach.

## Material and methods

### Identification and eligibility criteria of relevant studies

We conducted a PubMed (MEDLINE) and EMBASE search of the literature on the efficacy implications of combination therapy with ezetimibe and statin or double-dose statin on patients with hypercholesterolemia with the search strategies based on combinations of “statin”, “ezetimibe”, “double dose”, “low-density lipoprotein cholesterol (LDL-C)”, “total cholesterol (TC)”, “high-density lipoprotein cholesterol (HDL-C)”, and “hypercholesterolemia” from 2001 onward. Last query was updated on May 8, 2018. Reference sections of all retrieved articles were also screened to find out any studies missed.

Only double-blind Randomized controlled study (RCTs) of efficacy evaluation in the two groups with ezetimibe and statins and a double dose of statin in participants with hypercholesterolemia that examined low-density lipoprotein cholesterol (LDL-C), total cholesterol (TC) and high-density lipoprotein (HDL) were included. After read by two independent reviewers, the candidate articles were identified for the analysis studies based on title and abstract, which were both restricted to English. When cannot be categorized by the abstract, full-text review was retrieved. Reported data required for meta-analysis were then extracted. Studies with shorter or longer endpoint time (6~52 weeks) are excluded.

### Definitions and standardizations

All of the patients were randomly assigned to receive ezetimibe 10 mg and statin N mg (E/S) or statin 2 N mg (D/S). The efficacy of reducing LDL-C, TC and HDL-C concentrations in the two groups was recorded. All patients were assessed for LDL-C, TC and HDL-C level at end point time between the E/S and D/S treatment groups from baseline.

### Data extraction and risk of bias

The primary data were the level of LDL-C, TC and HDL-C concentrations at the end point and are expressed as mean and standard deviation (SD). Additional data obtained from the studies included publication year, the first author, age, number of male and total participants, *p* value, follow-up time and the dose of ezetimibe and statin. To ascertain the validity of eligible randomized trials, pairs of reviewers working independently and determined the adequacy of randomization and concealment of allocation, data collectors, and outcome assessors. The effect size between treatment groups within individual studies was assessed by weighted mean difference (MD). Disagreements were resolved by consensus between the two readers and studies included were all randomized double-blind controlled study.

### Data synthesis and meta-bias

Two reviewers worked on the data synthesis meta-bias of extracted data from all primary studies independently. All participants were classified in the E/S group or D/S group. A study was considered significant when the *p* value was less than 0.05 in univariate analysis. Heterogeneity was assessed for all endpoints with the I^2^ statistic. Considering the many sources of heterogeneity between studies and consequently between their individual MD, we calculated the overall MD according to the Der Simonian and Laird’s method [11], with a random effect model when homogeneity was not fine (*p*>0.10, I^2^ >50%) and a fixed effect model when I^2^ <50%. An observed higher negative MD indicated better cholesterol lowering effect for the treatment, with *p* < 0.05. RevMan 5.2 (Cochrane collaboration, Oxford, UK) was used for our analysis. Potential publication bias was evaluated by the Begg’s funnel plot and tested with STATA 11.0 (STATA Corporation, College Station, TX; X.L. M.). It was considered that there is no publication bias when the *p* value was more than 0.05 [12].

## Results

### Study selection

**Fig. 1:**
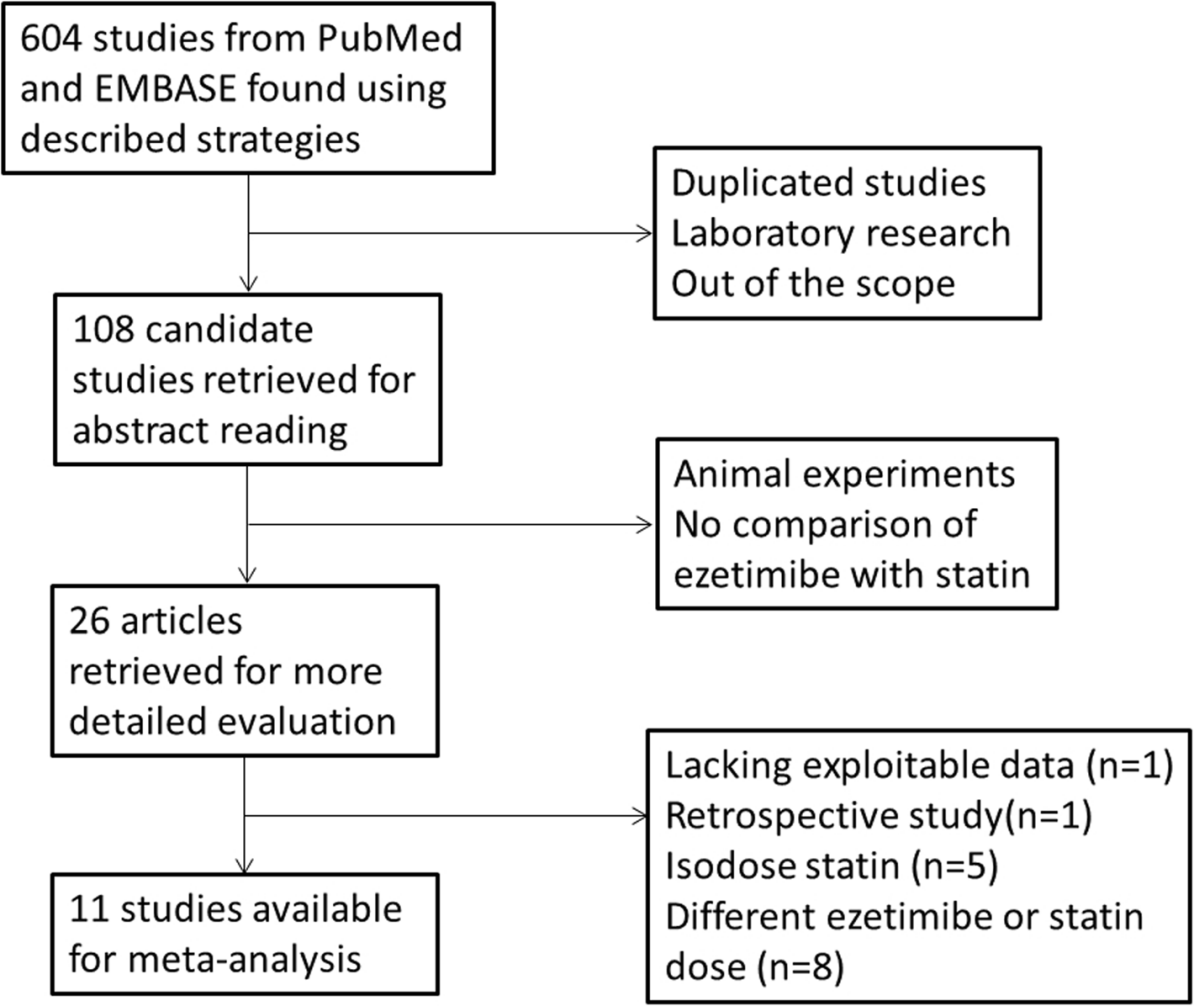
Selection of studies

Our electronic search algorithm retrieved a total of 604 initial citations for combination therapy with ezetimibe and statin or statin monotherapy and hypercholesterolemia. Following screening, 26 studies were identified for potential inclusion. Of these, 15 studies were excluded as lacking exploitable LDL-C, TC or HDL-C levels (*n* = 1), being a retrospective study (*n* = 1), comparing with isodose of statin (*n* = 5) and showing different ezetimibe or statin dose (*n* = 8). Finally, 11 studies (*n* = 1926 participants) were eligible for the meta-analysis (Fig. 1) [13–23].

### Study characteristics

Characteristics of the 11 eligible studies are listed in Table 1. Of all the 1926 participants with hypercholesterolemia, 994 (51.6%) participants received the combination therapy with ezetimibe and statin and 932(48.4%) received double-dose statin therapy alone. The mean age of the enrolled participants ranged from 56 to 70 years and the percentage of male is between 54 and 95%. No statistical difference was found between the two groups both in age and sex (*p*>0.05). Rosuvastatin (*n* = 8), simvastatin (*n* = 2), atorvastatin (*n* = 6) and pitavastatin (*n* = 2) were used in the included studies. 7 reports originated from Asia, 2 from Europe and 2 from America. The duration of study follow-up ranged from 6 to 52 weeks from baseline. All of the eligible studies were randomized double-blind controlled studies.

**Table 1:** Characteristics of the eligible studies

Study	Year	First Anthor	Country	Male	Age	Patients(n)		Therapy		follow-up	*p* value
				(%)	(Yrs)	E/S	D/S	EZE + Statin	Double Statin		
1	2018	Hong SJ	Korea	63/70	63/63	65	65	EZE 10 mg + ROS 5 mg	ROS 10 mg	8 week	< 0.001
				59/62	62/64	66	64	EZE 10 mg + ROS 10 mg	ROS 20 mg	8 week	< 0.001
2	2017	Ran D	China	76/73	60/60	42	41	EZE 10 mg + ROS 10 mg	ROS 20 mg	12 week	< 0.001
3	2017	Sakamoto K	Japan	NR	NR	51	53	EZE 10 mg + ATO 10 mg/PIT 1 mg	ATO 20 mg/PIT 2 mg	52 week	0.0002
4	2017	Japaridze L	Georgia	54/53	62/62	141	135	EZE 10 mg + ATO 20 mg/40 mg	ATO 40 mg/80 mg	16 week	< 0.001
5	2016	Farnier M	France	54/69	60/61	48	48	EZE 10 mg + ROS 10 mg	ROS 20 mg	12 week	NR
				59/72	63/60	53	53	EZE 10 mg + ROS 20 mg	ROS 40 mg	12 week	NR
6	2015	Sakamoto K	Japan	57/59	63/62	45	48	EZE 10 mg + ATO 10 mg/PIT 1 mg	ATO 20 mg/PIT 2 mg	12 week	< 0.001
7	2015	Saeedi R	Canada	95/85	56/57	21	18	EZE 10 mg + ROS 10 mg	ROS 20 mg	12 week	0.37
8	2015	Le NA	American	NR	64/64	133	74	EZE 10 mg + SIM 20 mg	SIM 40 mg	12 week	<0.01
9	2013	Matsue Y	Japan	72/75	69/70	117	133	EZE 10 mg + ATO 10 mg	ATO 20 mg	12 week	< 0.001
10	2012	Okada K	Japan	73/74	65/65	78	72	EZE 10 mg + ATO 10 mg/ROS 2.5 mg	ATO 20 mg/ROS 5 mg	12 week	<0.01
				73/74	65/65	78	72	EZE 10 mg + ATO 10 mg/ROS 2.5 mg	ATO 20 mg/ROS 5 mg	52 week	<0.01
11	2010	Averna M	Italy	54/57	61/62	56	56	EZE 10 mg + SIM 20 mg	SIM 40 mg	6 week	< 0.001

Data reported as Ezetimibe+Statin/Double-dose Statin(E/S, D/S)

Abbreviations: *EZE* Ezetimibe, *ROS* Rosuvastatin, *SIM* Simvastatin, *ATO* Atorvastatin, *PIT* Pitavastatin, *NR* Not reported

### Results and risk of bias

**Fig. 2:**
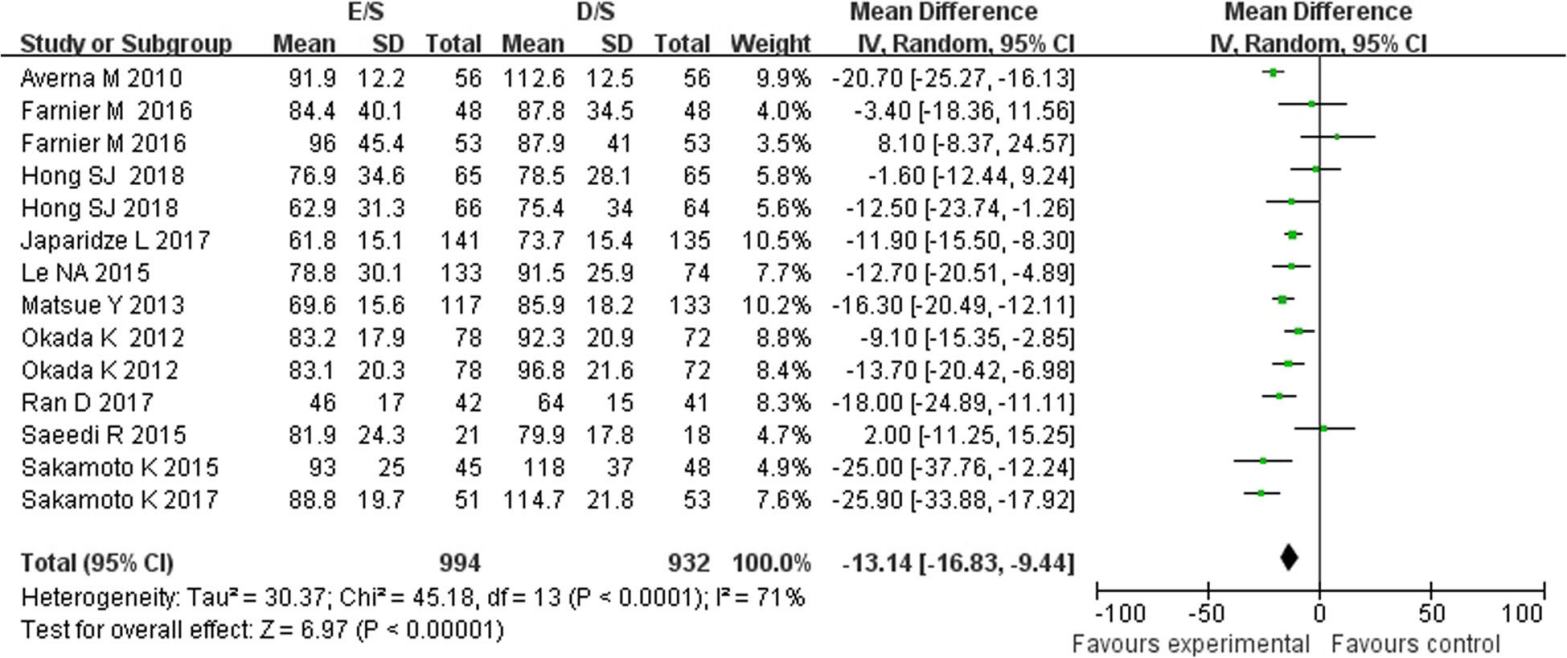
Meta-analysis of the change in LDL-C between groups [mg/dL]. Forest plot showing the effect of combined therapy with ezetimibe and statin versus double-dose statin only on plasma biomarkers of LDL-C; LDL-C, low-density lipoprotein cholesterol

**Fig. 3:**
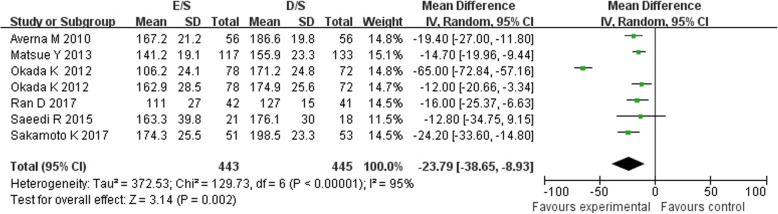
Meta-analysis of the change in TC between groups [mg/dL]. Forest plot showing the effect of combined therapy with ezetimibe and statin versus double-dose statin only on plasma biomarkers of TC; TC, total cholesterol

**Fig. 4:**
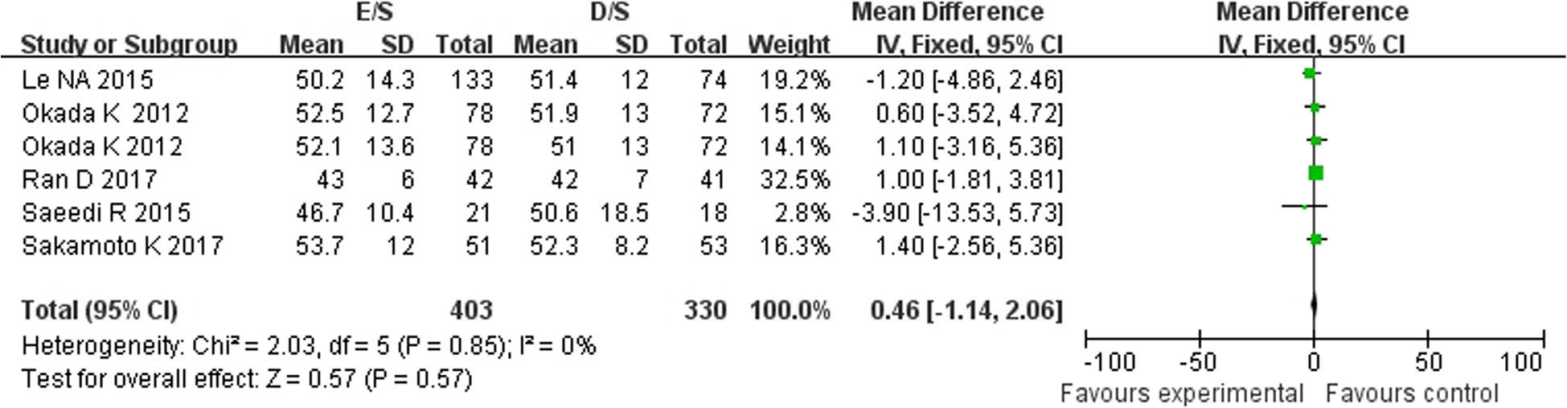
Meta-analysis of the change in HDL-C between groups [mg/dL]. Forest plot showing the effect of combined therapy with ezetimibe and statin versus double-dose statin only on plasma biomarkers of HDL-C; HDL-C, high-density lipoprotein cholesterol

**Fig. 5:**
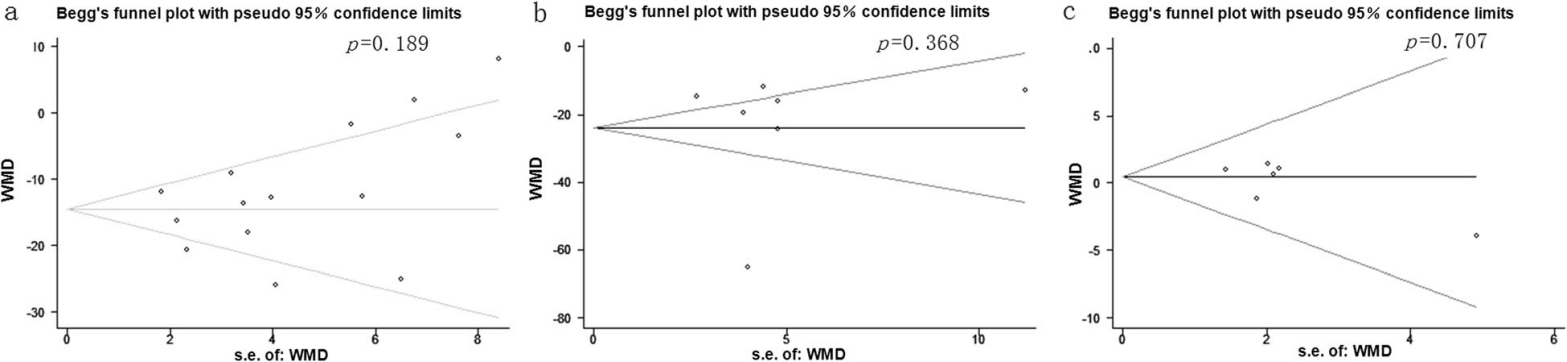
Funnel plots of publication bias summary for corresponding meta-analysis in (**a**, **b** and **c**)

Of the 11 included studies, 11 reported the data on LDL-C concentrations, 7 reported the TC and 6 reported the HDL-C, between baseline and follow up. Treatment with combination of ezetimibe and statin therapy associated with a significant lower LDL-C concentrations [MD = -13.14 mg/dL, 95%CI (− 16.83-9.44), *p* < 0.00001] when compared with double-dose statin therapy (Fig. 2). As between-study heterogeneity was significant (I^2^ = 71%, *p* < 0 .0001), random model was used. The patients in E/S group also got obvious lower TC concentrations [MD = -23.79 mg/dL, 95%CI (− 38.65-8.93), *p* = 0.002, I^2^ = 95%] from baseline to follow-up (Fig. 3). However, no significant between-group differences were observed for concentrations of HDL-C between treatment groups [MD = 0.46 mg/dL, 95%CI (−1.14, 2.06), *p* = 0.57, I^2^ = 0%] (Fig. 4). No significant publication biases were found in all results of meta-analyses according to Begg test (*p>0.05*) (Fig. 5a, b, c).

### Additional analysis

According to subgroup analysis of the 11 included studies (Fig. 2), the combination of ezetimibe and atorvastatin (10 mg) (Sakamoto K 2017, Sakamoto K 2015, Matsue Y 2013, Okada K 2012) [MD = -16.98 mg/dL, *p* < 0 .0001] or simvastatin (20 mg) (Le NA 2015, Averna M 2010) [MD = -17.35 mg/dL, *p* < 0 .0001] also showed stronger ability of reducing LDL-C, while the combination of ezetimibe and rosuvastatin (10 mg) (Hong SJ 2018, Ran D 2017, Farnier M 2016, Saeedi R 2015) [MD = -9.29 mg/dL, *p* = 0.05] showed less relevant. The efficacy of short-term [MD = -11.98 mg/dL, *p* < 0.00001] (endpoint time between 6 to 16 week, except Sakamoto K 2017 and Okada K 2012) and long-term (52 week, Sakamoto K 2017, Okada K 2012) [MD = -19.60 mg/dL, *p* = 0.001] treatment in the LDL-C between two groups did not show significant differences. Participants from Asia (Hong SJ 2018, Ran D 2017, Japaridze L 2017, Sakamoto K 2017, Sakamoto K 2015, Matsue Y 2013, Okada K 2012) treated with combination therapy were associated with a significant lower LDL-C concentration [MD = -14.7 mg/dL, *p* < 0 .0001]. However, no significant differences were found from the ones from Europe (Farnier M 2016, Averna M 2010) [MD = -6.61 mg/dL, *p* = 0.48] or America (Saeedi R 2015, Le NA 2015) [MD = -6.37 mg/dL, *p* = 0.38].

## Discussion

Plenty of studies have demonstrated that combination therapy with ezetimibe and statins have a significant LDL-C-lowering ability and cardiovascular events prevention effects. The primary findings of this study were that statin /ezetimibe 10 mg combination had a greater effect on lowering LDL-C and TC as compared to double-dose statin monotherapy.

The addition of ezetimibe to statin could produce an additive effect, improving the lipid profile markedly [24, 25]. Our results are consistent with this view. The overview and meta-analysis for the 11 primary studies revealed that both ezetimibe 10 mg plus statin and double-dose statin significantly reduced LDL-C and TC. According to the comparison, we found that combination therapy was associated with much greater reductions in circulating LDL-C and TC concentration levels, but no obvious changes in HDL-C concentration between treatment groups, suggesting that combination therapy should be a priority when statin resistance or poor efficacy happened.

Our subgroup analysis results also show differences. It was demonstrated that the combinations therapy with ezetimibe and different statins may have different ability to reduce cholesterol levels. According to the result, the combination of ezetimibe and atorvastatin (10 mg) or simvastatin (20 mg) showed stronger ability of reducing LDL-C, than rosuvastatin (10 mg). However, a previously trial results indicate that rosuvastatin (10 to 40 mg) has greater efficacy than atorvastatin (10 to 80 mg) or simvastatin (10 to 80 mg), for achievement of Adult Treatment Panel (ATP) III LDL-C goal of < 100 mg/dl (< 2.6 mmol/l) [26]. The findings in the current trial are converse. The variation may be related to different participants, addition of ezetimibe, too few related research and so on. Furthermore, the length of endpoint time of studies did not contribute to the difference, indicating that the combination therapy has a long-term and stable effect. The results also showed that only participants from Asia have stronger relevant of combination therapy and ability of reducing LDL-C, but comparing to Asia, too few studies (7 versus 2 versus 2) and participants (1366 versus 314 versus 246) from Europe and America were included. Of course, these variations may also have been attributed to genetic variation, compliance, time of administration and dietary intake. Therefore, more and further studies focusing on combination therapy with ezetimibe and statin and cholesterol levels are needed to verify our conclusions.

Between-study heterogeneity was significant in our analysis for LDL-C (I^2^ = 71% and TC (I^2^ = 95%), except the studies for the HDL-C evaluation (I^2^ = 0%). As heterogeneity was regarded as low, moderate or high based on an I^2^ value of 25, 50 and 75%, respectively [27], our analysis for LDL-C was considered moderate- heterogeneity and significant, but the TC analysis still need more verification. We tried to reduce the variability by screening the literature with the same standard and dividing studies into subgroups, such as a certain follow-up time, the same type of statin and the same geographic location of participant. Unfortunately, the heterogeneity could not be eliminated totally. But the heterogeneity decreased in some subgroups, such as combination with ezetimibe and rosuvastatin (I^2^ = 65%), atorvastatin (I^2^ = 69%), or simvastatin group (I^2^ = 67%), participants from Asia (I^2^ = 65%) and study with short follow-up time (endpoint time between 6 to 16 week) (I^2^ = 70%). This revealed that all the different factors have effects on the generation of heterogeneity which cannot be eliminated at the same time. Besides, all studies included are prospective randomized controlled studies, which are less prone to many biases than retrospective observational studies.

Some limitations of this meta-analysis should also be discussed. First of all, the number of samples in each study and even the total samples number (*n* = 1926) for meta-analysis is small. Moreover, the ability to reduce cholesterol levels by different statins may vary. Therefore, more and larger sample studies with the same statin are needed. In addition, we attempted to minimize publication bias by searching completely, but it is unavoidable that some data was missing for various reasons. Besides, results should be interpreted with caution. Disease state, accompanying disease, drug sensitivity difference and medication history may also contribute to its therapy efficacy.

## Conclusions

Although some modest bias cannot be excluded, this trial is the first study to evaluate and compare the efficacy of this combination therapy versus double-dose statin in patients. This meta-analysis revealed that the combination therapy with ezetimibe and statin appears to be more effective on reducing LDL-C and TC than doubling the statin dose. Future fundamental investigations and randomized controlled investigations with large samples are needed to confirm the efficacy of different statin in combination therapy for patients with hypercholesterolemia.

## Data Availability

Please contact author for data requests.

## Abbreviations

ACC/AHA: American Cardiology College/American Heart Association
ATP: Adult Treatment Panel
CHD: Coronary heart disease
D/S: Double dose of statin
E/S: Ezetimibe and statins
ESC/EAS: European Society of Cardiology/European Atherosclerotic Society
HDL: High-density lipoprotein
LDL-C: Low-density lipoprotein cholesterol
MD: Mean difference
NPC1L1: Niemann-Pick C1-like protein 1
PICOS: Participants, interventions, comparators, outcomes, and study design
SD: Standard deviation
TC: Total cholesterol
